# Evaluation of real‐world efficacy of mepolizumab on SNOT‐22 outcomes in patients with unified airway disease

**DOI:** 10.1002/clt2.70006

**Published:** 2024-10-31

**Authors:** Ruperto González‐Pérez, Paloma Poza‐Guedes, María Gabriela Martín‐Voso, Inmaculada Sánchez‐Machín

**Affiliations:** ^1^ Allergy Department Hospital Universitario de Canarias Santa Cruz de Tenerife Spain; ^2^ Severe Asthma Unit Hospital Universitario de Canarias Santa Cruz de Tenerife Spain; ^3^ Instituto de Investigación Sanitaria de Canarias (IISC) Santa Cruz de Tenerife Spain; ^4^ Allergy Department Hospital Universitario Puerta de Hierro Madrid Spain; ^5^ Immunotherapy Unit Hospital Universitario de Canarias Santa Cruz de Tenerife Spain

To the Editor,

The unified airway hypothesis suggests that the upper and lower airways constitute a single, interconnected organ, sharing significant physiological traits such as immunology, pathophysiology, epidemiology, and clinical features.^1^ Allergic rhinitis (AR), various forms of chronic rhinosinusitis (CRS), including those with nasal polyposis (NP), and severe asthma (SA) share a common underlying pathogenesis known as type‐2 inflammation (T2).^2^ These coexisting conditions represent a substantial clinical burden and significantly impact patients' quality of life (QoL) through a variety of disruptive symptoms The SNOT‐22 is a validated questionary designed to assess the symptom burden of CRS and has proven effective utility in evaluating both QoL and symptom control in AR.^3^, ^4^

Mepolizumab, a monthly subcutaneous IL‐5 antagonist monoclonal antibody, has been approved for treating various type 2 inflammatory conditions, including both eosinophilic SA and CRSwNP.^5^, ^6^ The performance of mepolizumab on SNOT‐22 scores has not been completely assessed in patients afflicted with Unified Airway Disease (UAD).

This Phase IV, single‐center observational cohort investigation, conducted at Hospital Universitario de Canarias in Tenerife, Spain, aimed to evaluate the real‐world performance of mepolizumab 100 mg every 4 weeks (100 mg‐q4w) over 52‐weeks on SNOT‐22 scores in patients aged over 18 years with UAD, including uncontrolled SA and persistent mite (*Dermatophagoides spp*. and/or *Blomia tropicalis*) AR, with or without comorbid CRSwNP. Key inclusion criteria were a clinician‐confirmed diagnosis of SA and AR with a T2 signature according to specific guidelines^7^, ^8^ and a CRSwNP diagnosis based on EPOS criteria.^9^ Pregnant and breast‐feeding women were excluded. The study was approved by the local Ethical Committee of our Institution and informed consent was signed by all participants.

Data from clinical records collected between January 2021 and July 2024 were retrospectively analyzed, with a total of 102 patients screened. Among them, 71 patients—40 females and 31 males, median age 48 years (IQR 21)—were confirmed as eligible for the study (Table S1). Outcome data from all participants were gathered and compared at two time points: before (T0) and 52‐weeks post‐commencement (T1) of monthly mepolizumab. Quantitative variables were presented as median and interquartile range (IQR), whereas qualitative variables were expressed as number of observations and percentage. Individual SNOT‐22 item scores were summed, and a median domain score was derived. Pulmonary function test, including pre‐ and post‐bronchodilator spirometry (Datospir 600^®^, Sibel S.A.U., Barcelona, Spain) and a validated asthma control test (ACT) were conducted in accordance with daily practice guidelines. Comparison of findings between baseline and different follow‐ups were performed using the Wilcoxon matched pairs signed rank and paired *t* tests. A *p* value of less than 0.05 was considered statistically significant. All statistical data were analyzed using GraphPad Prism version 10.2.3 for Windows, GraphPad Software, La Jolla, CA, USA.

In the present study (Table 1), patients with CRSwNP exhibited a significantly higher median baseline SNOT‐22 score compared to those without CRSwNP (57 (22) versus 38 (36), *p* = 0.0136). After 52 weeks of treatment with mepolizumab 100 mg mg‐q4w, patients with CRSwNP showed a marked median reduction in their SNOT‐22 score (from 58 (22) to 32 (32.25), *p* = 0.0001), whereas the improvement in patients without CRSwNP was not significant (from 38 (36) to 36 (29), *p* = 0.1126) (Figure 1). Furthermore, a clinically significant improvement (more than 8.9 points) in the overall SNOT‐22 score was observed in 25 out of 35 individuals from the CRSwNP group compared to 8 out of 36 patients in the non‐CRSwNP group. Interestingly, 21 of the 22 SNOT‐22 items ‐excluding “Embarrassed” from the emotional domain‐significantly improved (*p* < 0.05) after 52 weeks of mepolizumab 100 mg‐q4w in patients with CRSwNP, unlike those without this comorbidity. Notably, while all items in the SNOT‐22 nasal domain significantly enhanced only in patients with CRSwNP, the specific item “Decreased sense of smell/taste” showed significant improvement in both patients with (*p* = 0.002) and without CRSwNP (*p* = 0.012). Regarding T2‐inflammation biomarkers a significant reduction (*p* < 0.0001) in the absolute blood eosinophil counts after 52 weeks of treatment with mepolizumab was observed with no significant changes recorded in total IgE, specific IgE (sIgE) to dust mites, or fractional exhaled nitric oxide (FeNO) in either study group (Table S2).

**TABLE 1:** Assessment of SNOT‐22 outcomes in patients with unified airway disease (*n* = 71) at baseline (T0) and after 52‐week (T1) treatment with subcutaneous mepolizumab 100 mg every 4 weeks.

Variables	SA&AR&CRSwNP T0	SA&AR&CRSwNP T1	SA&AR T0	SA&AR T1
SNOT‐22 total score (scale 0–110)	57 (22)	32 (32.25)*	38 (36)	36 (29)
Nasal domain				
Need to blow nose SNOT‐22 (scale 0–5)	4 (2)	2 (2.25)*	2 (2)	2 (2)
Nasal blockage (scale 0–5)	4 (1.25)	2 (3)*	2 (2)	2 (2)
Sneezing (scale 0–5)	2 (3)	1 (2.25)*	2 (2)	2 (2)
Runny nose (scale 0–5)	4 (2)	2 (2)*	2 (2)	2 (2)
Cough (scale 0–5)	2 (3)	1 (2)*	2 (3)	2 (2)
Post‐nasal discharge (scale 0–5)	4 (2.25)	1.5 (4)*	1 (3)	1 (2)
Thick nasal discharge (scale 0–5)	4 (3)	2 (2.25)*	1 (2)	0.5 (3)
Decreased sense of smell/taste (scale 0–5)	5 (1)	4 (2.5)*	1 (3)	0 (1)*
Ear/facial domain				
Ear fullness (scale 0–5)	4 (2)	1 (3.25)*	2 (4)	0 (3)
Dizziness (scale 0–5)	1 (2)	0 (1)*	0 (2)	0 (1)
Ear pain (scale 0–5)	1 (2)	0 (2)*	0 (2)	0 (2)
Facial pain/pressure (scale 0–5)	3 (5)	0.58 (2)*	1 (2)	1 (3)
Sleep domain				
Difficulty falling asleep (scale 0–5)	2 (2.25)	1 (2)*	2 (3)	2 (3)
Wake up at night* (scale 0–5)	2 (2)	1 (2)*	2 (3)	2 (3)
Lack of a good night's sleep (scale 0–5)	2.5 (3)	1 (3)*	2 (3)	1 (3)
Wake up tired* (scale 0–5)	3 (2)	1 (3)*	2 (2)	1 (4)
Function domain				
Fatigue (scale 0–5)	3 (2.25)	1 (3)*	2 (2)	2 (3)
Reduced productivity (scale 0–5)	3 (3)	1 (2.25)*	2 (2)	2 (3)
Reduced concentration (scale 0–5)	2 (3.25)	1 (3)*	2 (3)	2 (3)
Emotion domain				
Frustrated/restless/irritable (scale 0–5)	3 (3.25)	1 (3)*	2 (2)	1 (3)
Sad* (scale 0–5)	2 (3)	0 (2)*	1 (3)	1 (2)
Embarrassed (scale 0–5)	5 (2)	0 (1)	0 (3)	0 (1)

*Note*: Median values and interquartile range are shown. (*) Indicates statistical significance (*p* < 0.05).

**FIGURE 1:**
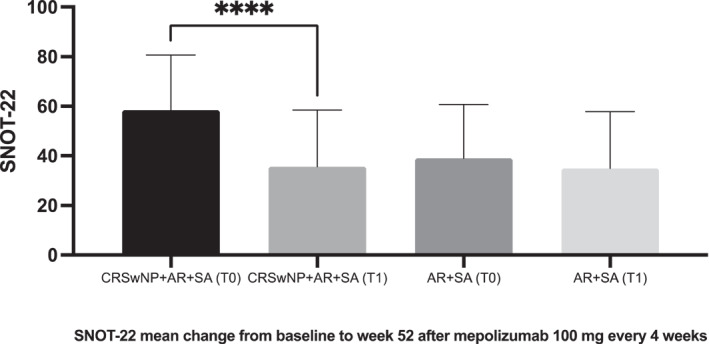
Evolution of Sino‐Nasal Sino‐Nasal Outcome Test‐22 (SNOT‐22) score in patients (*n* = 71) with T2 unified airways disease, including uncontrolled severe asthma (SA) and persistent allergic rhinitis (AR) with or without chronic rhinosinusitis with nasal polyps (CRSwNP) at baseline (T0) and after and 52‐week mepolizumab 100 mg every 4 weeks (T1). Mean values and standard deviation are shown. (****) Indicates statistical significance (*p* < 0.0001).

Due to a lack of improvement in sino‐nasal symptoms, two out of 35 (5.71%) patients with CRSwNP required functional endoscopic sinus surgery (FESS) after 24–32 weeks of mepolizumab 100 mg‐q4w, continuing the treatment post‐surgery. No patients experienced adverse events leading to mepolizumab discontinuation throughout the study period. The size of the investigated population, and the lack of a control group should be considered potential limitations of this investigation when interpreting the overall data.

In conclusion, these findings demonstrate the long‐term real‐world effectiveness of mepolizumab 100 mg‐q4w in enhancing all SNOT‐22 domains in a selected cohort of patients with UAD. Our results confirmed that mepolizumab significantly reduces symptom severity and improves overall QoL, particularly in patients with UAD, irrespective of comorbid NERD, prior surgery, and/or systemic corticosteroid use in a specifically selected population. The interconnection between upper and lower airway T2‐inflammation diseases highlights the need for a more holistic approach to patient assessment and management. Further research is essential to better understand the synergistic effects of precision therapy, optimize outcomes for UAD patients extrapolated from other populations, and ensure the safety of drug interventions.

## AUTHOR CONTRIBUTIONS

**Ruperto González‐Pérez**: Conceptualization; investigation; funding acquisition; writing—original draft; methodology; writing—review and editing; data curation; resources. **Paloma Poza‐Guedes**: Conceptualization; investigation; methodology; writing—review and editing; software; project administration; data curation; supervision. **María Gabriela Martín‐Voso**: Investigation; data curation; visualization; software. **Inmaculada Sánchez‐Machín**: Supervision; resources; validation; writing—review and editing.

## CONFLICT OF INTEREST STATEMENT

The authors declare no conflict of interest. The funders had no role in the design of the study; in the collection, analyses, or interpretation of data; in the writing of the manuscript, or in the decision to publish the results.

## Supporting information

Supporting Information S1
