# Supplementary material for: Evaluation of real‐world efficacy of mepolizumab on SNOT‐22 outcomes in patients with unified airway disease
Source: Clin Transl Allergy. 2024 Oct 31;14(11):e70006. doi: 10.1002/clt2.70006 (PMC11527812; doi:10.1002/clt2.70006)
Supplement: Supplementary file 1 — Supporting Information S1 [file CLT2-14-e70006-s001.doc]

Supplementary table S1

**Table S1:** Demographic and clinical characteristics of patients before commencement of mepolizumab.

| **Demographic variables (n=71)** | SA&AR&CRSwNP | SA&AR |
| --- | --- | --- |
| n (%) | 35 (54.68) | 36 (45.32) |
| Age (y), median (IQR) | 51 (13) | 47 (30) |
| Sex distribution (F/M) | (18/17) | (21/15) |
| BMI | 27.32 ± 3.15 | 28.12 ± 4.21 |
| Asthma onset at childhood, n (%) | 29 (82.85) | 23 (79.31) |
| Family history of atopy, n (%) | 32 (91.42) | 26 (89.65) |
| NERD, n (%) | 11 (31.2) | 0 (0) |
| Any prior FEES, n (%) | 32 (91.42) | 0 (0) |
| Use of ≥2 SCS in the previous y n (%) | 35 (100) | 29 (100) |
| Prior use of biologics (SA indication) | 7 (20) | 8 (22.22) |
| ACT | 15.22 ± 4.62 | 14.34 ± 3.71 |
| FVC, mL (%) | 3,610 (88.2) | 3,360 (78.41) |
| FEV1, mL (%) | 2,440 (76.36) | 2,280 (73.41) |

Median values and interquartile range are shown. SA: Severe asthma. AR: Allergic Rhinitis. CRSwNP: Chronic rhinosinusitis with nasal polyps. NERD: NSAID-exacerbated respiratory disease. FEES: Functional endoscopic sinus surgery. SCS: Systemic corticosteroids. ACT: Asthma Control Test. FVC: Forced Ventilatory Capacity. FEV1: Forced expiratory volume in the ﬁrst second.

Supplementary table S2

**Table S2:** Analysis of type 2-inflammation biomarkers in patients with unified airway disease (*n* = 71) at baseline (T0) and after 52-week (T1) treatment with subcutaneous mepolizumab 100 mg every 4 weeks.

| T2-inflammation Biomarker | SA&AR&CRSwNP T0 | SA&AR&CRSwNP T1 | SA&AR T0 | SA&AR T1 |
| --- | --- | --- | --- | --- |
| Eosinophils/µL peripheral blood | 572 ± 335 | 58.26 ± 48.28* | 484.82 ± 248.11 | 71.85 ± 52.18* |
| Total IgE (IU/mL) | 469.5 ± 553 | 367 ± 419 | 675.09 ± 969.84 | 620 ± 1,105 |
| sIgE *D. pteronyssinus* (kUA/L) | 21.05 ± 49.21 | 18.61 ± 32.35 | 40.69 ± 40.99 | 34.2 ± 38.94 |
| sIgE *D. farinae* (kUA/L) | 10.74 ± 20.13 | 12.37 ± 26.9 | 36.58 ± 41.38 | 33.32 ± 35.46 |
| sIgE *Blomia tropicalis* (kUA/L) | 1.96 ± 7.31 | 1.98 ± 7.73 | 3.83 ± 6.51 | 3.17 ± 6.48 |
| FENO (ppb) | 64.53 ± 52.35 | 68.02 ± 59.44 | 49.33 ± 32.11 | 46.66 ± 24.41 |
|  |  |  |  |  |

Mean values and standard deviation are shown. SA: Severe asthma. AR: Allergic Rhinitis. CRSwNP: Chronic rhinosinusitis with nasal polyps. sIgE: Speciﬁc IgE. *D. pteronyssinus*: *Dermatophagoides pteronyssinus*. *D*. *farinae*: *Dermatophagoides farinae*. FENO: Fractional exhaled nitric oxide. (*) Indicates statistical signiﬁcance (p < 0.05).
